# Intravitreous delivery of melatonin affects the retinal neuron survival and visual signal transmission: *in vivo* and *ex vivo* study

**DOI:** 10.1080/10717544.2020.1818882

**Published:** 2020-10-05

**Authors:** Ye Tao, Bang Hu, Zhao Ma, Haijun Li, Enming Du, Gang Wang, Biao Xing, Jie Ma, Zongming Song

**Affiliations:** a Department of Ophthalmology, People’s hospital of Zhengzhou University, Zhengzhou, PR China; b Department of physiology and neuroscience, Basic college of medicine, Zhengzhou University Zhengzhou, PR China; c Department of Colorectal Surgery, The Sixth Affiliated Hospital, Sun Yat-sen University, Guangzhou, PR China; d Department of Neurosurgery, Central Hospital of Wuhan, Tongji Medical College, Huazhong University of Science and Technology, Wu Han, PR China

**Keywords:** Medicine delivery, neurodegeneration, therapeutics, intravitreous

## Abstract

Intravitreal delivery can maximize the intensity of therapeutic agents and extend their residence time within ocular tissue. Melatonin is a lipophilic molecule that crosses freely biological barriers and cell membranes. This study intends to investigate the effects of intravitreally delivered melatonin on mouse retina. The visual function of administered mice is assessed by electrophysiological and behavior examinations three weeks after intravitreal delivery. Moreover, multi-electrode array (MEA) was used to assess the electrical activities of retinal ganglion cells (RGCs). We found that intravitreal delivery of high dosage melatonin (400–500 µg/kg) destroyed the retinal architecture and impaired the visual function of mice. Conversely, the melatonin administration at low dose (100–300 µg/kg) did not have any significant effects on the photoreceptor survival or visual function. As shown in the MEA recording, the photoreceptors activity of the central region was more severely disturbed by the high dose melatonin. A pronounced augment of the spontaneous firing frequency was recorded in these mice received high dosage melatonin, indicating that intravitreal delivery of high dosage melatonin would affect the electrical activity of RGCs. Immunostaining assay showed that the vitality of cone photoreceptor was impaired by high dose melatonin. These findings suggest that intravitreal melatonin is not always beneficial for ocular tissues, especially when it is administered at high dosage. These data add new perspectives to current knowledge about melatonin delivery at the ocular level. Further therapeutic strategies should take into consideration of these risks that caused by delivery approach.

## Introduction

Eye is a complex organ with multi-compartmental structure and unique anatomy. The transparent anterior segment of eyeball would allow for the minimally invasive drug delivery and sequential in vivo evaluations (Constable et al., 2017; Garafalo et al., 2019). On the other hand, the posterior segment of eye is protected by consolidated static and dynamic barriers which would restrict the drug transportation to the target region. Due to the complicated physiology of these barriers, the posterior segment ocular diseases, such as retinopathy and optic neuropathy, present enormous challenges for pharmacological therapeutics (Varela-Fernández et al., 2020). It is imperative to develop efficient delivery strategy and supporting materials for these diseases affecting the posterior segment. Intravitreal injection is an effective delivery approach to maximize the concentration of therapeutic agents in the posterior cavity, allowing longer residence time and release course within the eye (Nayak & Misra, 2018; Cabrera et al., 2019). After delivered into the vitreous, the pharmacological agents would contact directly with the retina, minimizing the side-effects that commonly seen in systemic delivery. Thus far, the intravitreal delivery of pharmacological agents has been used for treatment of retinopathies and vitreous diseases. Diverse therapeutic molecules are delivered intravitreally in ophthalmological practice, including the viral vectors, peptides, proteins, and nucleic acids (Maier et al., 2013; Hartman & Kompella, 2018).

Melatonin is neurohormone that regulates circadian rhythm *via* autocrine or paracrine manner. A pronounced level of melatonin is detected in the aqueous humor and retinal tissue (Felder-Schmittbuhl et al., 2018). In the eye, melatonin molecules create a concentration gradient, which in turn drives them to diffuse across the internal limiting membrane, and touch the retinal neurons directly. Melatonin receptor subtypes MT1 and MT2 are expressed in several laminations of retina, including the inner nuclear layer, the inner plexiform layer, RGCs layer, and retinal pigmented epithelium layer (Wiechmann & Sherry, 2013). Melatonin influences numerous ocular functions, such as the modulating light sensitivity, controlling neurotransmitter release, and adjusting intraocular pressure (Ostrin, 2019). On the other hand, solid evidences have shown that melatonin acts as a robust scavenger of reactive oxygen species (ROS) under pathological conditions (Cosker et al., 2020). Its administration can also stop, decrease, or even inhibit some of crucial steps of apoptotic cascades. In this context, MEL is proposed as a candidate pharmacotherapy for its antioxidant and anti-apoptotic characteristics. Clinical and animal researches show that melatonin protects against the oxidative damages of various ocular disease (McMahon et al., 2014). These beneficial data highlight the possibility that melatonin might be developed as a promising medication for ocular diseases.

Exogenous melatonin is generally administered through the systemic or topical routes in clinical and experimental trials. Recent studies show that the intravitreal delivery of melatonin could also be beneficial for retinal neurons (García-Caballero et al., 2018). Intravitreally delivered melatonin alleviates the retinal ganglion cells (RGCs) death in a rabbit model with glaucomatous pathology (Arranz-Romera et al., 2019). Intravitreal delivery of melatonin also alleviates the photoreceptor apoptosis and preserves the visual signal pathway in a mice model with retinal degeneration (Li et al., 2019). This delivery approach not only ensures higher melatonin concentration in the targeted tissue, but also avoids side effects on these unwanted organs. These advantages would lay the groundwork for further intraocular application of melatonin. However, the pharmacokinetic and biopharmaceutics aspects for the intravitreal delivery of melatonin remain unknown. Exogenous melatonin is not always beneficial, since it can increase the light susceptibility, and induce photoreceptor disruption under high-intensity illumination (Sugawara et al., 1998). The melatonin is generally delivered *via* systemic routes in pharmacological experiments. Intravitreal delivery is uncommon and only several reports are available. A previous study shows that intravitreal injection of melatonin at the dose of 200 µg/kg produced severe morphological damages such as RGCs death and formation of retinal edema, indicating that high dose of intravitreal melatonin may have toxic effects on retina (Yilmaz et al., 2004). On the other hand, intravitreal injection of melatonin at doses ranging from 50 to150 µg/kg does not produce any morphological changes. Furthermore, another study shows that intravitreal injection of melatonin at doses ranging from 50 to 150 µg/kg is able to ameliorate the N-methyl-N-nitrosourea (MNU) induced photoreceptor apoptosis (Li et al., 2019), suggesting that intravitreal injection of melatonin at rational doses (50–150 µg/kg) would be therapeutic. Thus clarifying the toxic effects and safe dose of intravitreal melatonin is of special importance. This study intends to investigate the effects of intravitreally delivered melatonin on the viability of retinal neurons. Furthermore, the visual function of these administered mice is monitored by the multi electrode array (MEA) recording. Our results highlight that high dose of melatonin would cause morphological and functional impairments to retinas when they are delivered intravitreally. Further therapeutic strategies should take into consideration of the potential risks that caused by delivery approaches.

## Materials and method

### Animal handling

Animals were handled following the ARVO guidelines for the Use of Animals in Ophthalmic and Vision Research. Totally 320 mice (C57/BL, 8 weeks old with both sexes, each weighing between 19 and 23 g) were housed in the specific pathogen-free facility (18–23 °C, 40–65% humidity) with food and water available ad libitum. All the mice were housed at the same light level. The illumination in the animal facility was 85 lux (12 h dark/light cycle with lights on at 07:00 and off at 19:00). Melatonin (Sigma-Aldrich Corp; St. Louis, MO) was initially dissolved in 5% DMSO and then further diluted with PBS at various concentrations (Andrés-Guerrero et al., 2009; Berger et al., 2017). Control animals received vehicle injection containing the same amount of DMSO and PBS as given to the melatonin treated groups. Mouse of each group was subjective to an intravitreal injection of melatonin at the dose of 100, 200, 300, 400, and 500 μg/kg, respectively. All the intravitreal injections of melatonin were performed at 8:00PM. The doses of melatonin selected in this study were based on data from other pharmacological investigators that delivered melatonin via intravitreal route (Yilmaz et al., 2004; García-Caballero et al., 2018).

### Intravitreal delivery

The mixture of ketamine (80 mg/kg BW) and chlorpromazine (8 mg/kg BW) was injected intraperitoneally to anesthetize the animal. Atropine (1%) and phenylephrine hydrochloride (2.5%) eye drops (Xing Qi, Shenyang, China) were applied topically to dilate the pupils. The syringe needle of a 30-gauge microinjector was inserted through sclerocorneal limbus at a 45°angle into the vitreous body. Subsequently, the solution was injected into the midvitreous cavity within 30 s. Immediately after removing the syringe needle, the neomycin eye ointment was used to prevent bacterial infection. These injected eyes were examined with an ophthalmoscope every day. Three weeks after delivery, these mice received functional and morphological analysis.

### Optokinetic behavioral test

Three weeks after intravitreal delivery, the optokinetic behavioral tests were performed to analyze the visual function of animals. A two-alternative forced-choice paradigm was used to analyze the optomotor responses to moving sine wave gratings (McGill et al., 2012). Mouse was placed on a pedestal situated in the center of a square array (OptoMotry CerebralMechanics, Lethbridge, AB, Canada). Stimulus gratings were projected on the wall of the machine and they turned randomly in clockwise or counter-clockwise direction. Mice reflexively track the rotating virtual cylinders by moving their head in the direction of rotating gratings. An infrared television camera located at the top of the testing machine allowed the examiner to decide the direction of animal’s optomotor response. The initial stimulus was set as 0.200 cyc/deg sinusoidal pattern at a fixed 100% contrast. The contrast sensitivity was measured at spatial frequencies ranging between 0.014 and 0.511 cyc/deg.

### Electroretinogram (ERG) examination

After the optokinetic behavioral tests, mice were dark adapted for 12 h and were anesthetized under dim red illumination. Their pupils were dilated and then they were transferred to the recording plane of a RETIport system (Roland Consult, Germany). Platinum circellus electrodes were placed in contact with the central cornea and a reference electrode was inserted subcutaneously into the forehead. The scotopic responses of animals were stimulated using while light at the intensity of 0.5 log cd-s/m^2^. Photopic recordings were made at the intensity level of 1.48 log cd-s/m^2^. Signals were amplified and filtered by the band pass (1–300 Hz). For each animal, 60 photopic and 10 scotopic stimuli were applied and the average value of the records was registered. For waveform analysis, the amplitude of a-wave was defined as the distance from baseline to a-wave trough, while the amplitude of b-wave was measured as the distance between trough and peak of each waveform. All the ERG examinations were performed at the same time of the day to void circadian variation.

## Spectral-domain optical coherence tomography (SD-OCT)

Three weeks after intravitreal delivery, these mice subjected to morphological analysis. Retinal SD-OCT images of the anesthetized animals were obtained with the Micron IV imaging system ultrahigh-resolution instrument Micron IV imaging instrument (Phoenix Research, Pleasanton, CA). The lubricant gel (Allergan Inc., Dublin, Ireland) was applied to the eye surface before imaging. A probe was placed in front of the cornea, and the position was adjusted until high-resolution images appeared on the monitor screen. The mice were subjected to repeated dilations before examination. The measurements were made of a linearized box with a diameter of 0.3 mm centered on the optic nerve head (ONH). The retinal thickness was calculated using computing software kit.

### Multi-electrode array (MEA) recording

MEA recording was carried out using the MED-64 system (Alpha med Sciences, Osaka, Japan) (Ye et al., 2015). Retinal specimens were placed in connected with the electrodes array of recording chamber. White light (with the intensity of 850 mcd-s/m^2^) from a stimulator was casted onto the retinal specimens to elicit the local field potentials (LFPs). The electrical physiological signals of retinal neurons were collected by the electrodes, and then transmitted into the monitor for process. The firing action potentials of RGCs were sorted out with offline software and filtered with a band pass (100–3000 Hz). The post-stimulus time histogram (PSTH) and the raster plots of individual units were used to categorize the light-induced responses of RGCs. The interspike interval (ISI) histograms analysis was used to evaluate the discharge patterns of the spontaneous firing response, thereby classifying the firing pattern of RGCs.

### Istological and immunohistochemical analysis

The eyeballs of experimental animals were collected and fixed 4% paraformaldehyde for 4 h at 4 °C. The retinal specimens were dehydrated in 30% glucose solution for 12 h and then embedded in optimum cutting temperature (OCT) compound. Frozen sections were cut through the ONH and then were stained with hematoxylin-eosin for visualizing sections. The outer nucleus layer (ONL) and the number of cells in the ganglion cell layer (GCL) were calculated as described previously (Ye et al., 2015). The thicknesses of the ONL were measured at 250 µm intervals along the vertically superior–inferior axis by a single observer (Sakami et al., 2011). For immunohistochemistry, the retinal specimens (sections and whole mounts) were blocked with 3% H_2_O_2_ deionized water at 37 °C for 30 min, and were incubated subsequently with primary antibodies (anti-cone opsin,1:400, Millipore, Billerica, MA) or peanut agglutinin (PNA) conjugated to a Alexa Fluor 488 (1:200, Invitrogen, Carlsbad, CA) overnight at 4 °C. The retinal specimens were rinsed thoroughly, and then were incubated with Cy3-conjugated anti-rabbit IgG (1:400, Jackson ImmunoResearch Laboratories, West Grove, PA) and 4′,6 -diamidino-2-phenylindole-dihydrochloride (DAPI, D9542; Sigma-Aldrich, Natick, MA) at 37 °C for 30 min. Cone numbers within four 420 × 420 μm bins located 1 mm dorsal, temporal, ventral, and nasal to the ONL were calculated using Axiovision Rel. soft.

### Statistical analysis

Statistical differences were processed with the ANOVA analysis followed by Bonferroni’s *post-hoc* analysis. All data are presented as mean ± standard deviation (SD). *p* Value <.05 was considered to be significant.

## Results

### Morphological changes after intravitreal delivery of melatonin

**Figure 1.**
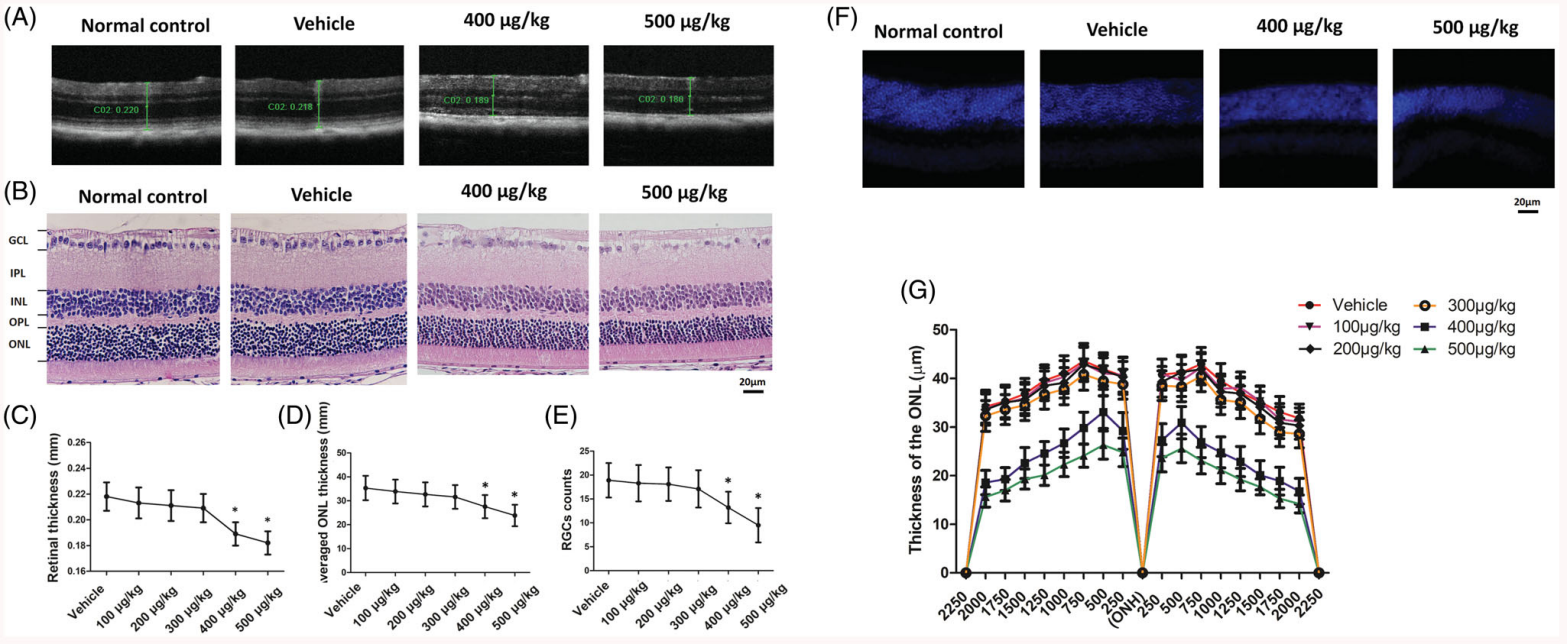
(A) OCT assary showed that the retinal architectures in the 400 and 500 µg/kg groups were destroyed severely after melatonin delivery. (B) Histological assay found that the ONL in the 400 and 500 µg/kg groups became thinner compared with normal controls. The mean retinal thickness (C) and ONL thickness (D) in the 400 and 500 µg/kg group reduced significantly in the 400 and 500 µg/kg groups. (E) Less cells were detected in the GCL of retinal sections in 400 and 500 µg/kg groups. (F) The nucleus in the ONL of the 400 and 500 µg/kg groups was much less compared with normal controls. (G) The ONL thickness of the 400 and 500 µg/kg groups was ubiquitously smaller than the normal controls in different measuring points (ANOVA analysis followed by Bonferroni’s *post hoc* analysis was performed, *p <  .05, for differences between groups).

No intraocular hemorrhage occurred during the melatonin delivery. The lenses of these mice were transparent after intravitreal injection. No macrophage or monocyte infiltration was detected in the anterior chamber or vitreous of these animals. OCT measurement showed that the retinal architectures were intact and consolidated in vehicle, 100, 200, and 300 µg/kg groups. Conversely, the retinal architectures in the 400 and 500 µg/kg groups were destroyed severely after melatonin delivery (Figure 1(A)). OCT examination showed that the retina thickness in the 400 and 500 µg/kg groups declined significantly compared with normal controls (*p* < .01; *n* = 10; Figure 1(C)). Histological assay found that the mean ONL thickness declined significantly in the 400 and 500 µg/kg groups (*p* < .01; *n*= 10; Figure 1(B)). The mean ONL thickness in the 400 and 500 µg/kg group was ∼76.9% and ∼66.6% of the normal controls, respectively. The mean ONL thickness was not significantly different between the vehicle, 100, 200, 300 µg/kg and the normal control group (*p* > .05; *n* = 10; Figure 1(D)).

**Figure 2.**
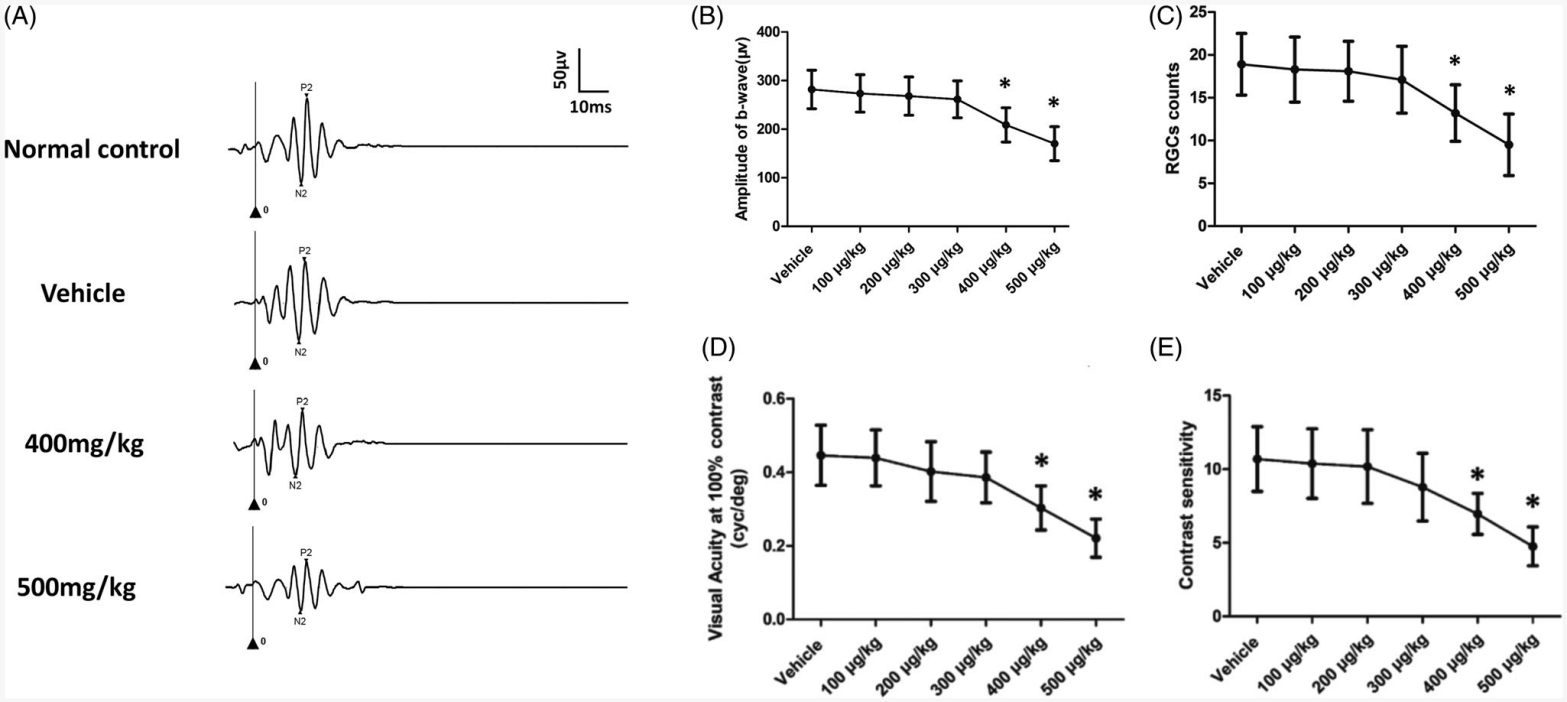
(A) ERG examinations showed that the oscillatory potentials (OPs) waveforms of the 400 and 500 µg/kg groups were affected after melatonin delivery. (B) The OPs amplitudes reduced significantly in the 400 and 500 µg/kg groups compared with that in normal control group. Optokinetic behavioral tests were performed to evaluate the status of visual function. The visual acuity (D) and contrast sensitivity (E) of the 400 and 500 µg/kg groups declined significantly compared with normal control, suggesting that intravitreal melatonin at high dosage yielded deleterious effects on the optokinetic response (ANOVA analysis followed by Bonferroni’s *post hoc* analysis was performed, *p< .05, for differences between groups).

The nucleus in the ONL of the 400 and 500 µg/kg groups was much less compared with normal controls (Figure 1(F)). The photoreceptors in this ONL were tightly and regularly arranged. The number of cells in the GCL also reduced significantly in the 400 and 500 µg/kg groups (*p* < .01; *n* = 10; Figure 1(E)). The number of cells in the GCL in the 400 and 500 µg/kg group was, respectively, ∼69.8% and ∼50.2% of the normal controls. Furthermore, the adjacent thickness of the ONL was measured along the vertically superior–inferior axis. The ONL thickness of the 400 and 500 µg/kg groups were ubiquitously smaller than the normal controls in different points (Figure 1(G)). These results showed that the intravitreal melatonin at high dosage induced toxic effects on the retinal structure. Moreover, ERG examinations were carried out to analyze the oscillatory potentials (OPs) of these mice (Figure 2(A)). The OPs amplitudes reduced significantly in the 400 and 500 µg/kg groups than that in normal control group (*p* < .01; *n* = 10; Figure 2(B)). The mean OPs amplitude in the 400 and 500 µg/kg group was, respectively, ∼73.2% and ∼53.8% of the normal controls, indicating that intravitreal melatonin at high dosage would affect the visual function of RGCs.

### Functional changes after intravitreal delivery of melatonin

**Figure 3.**
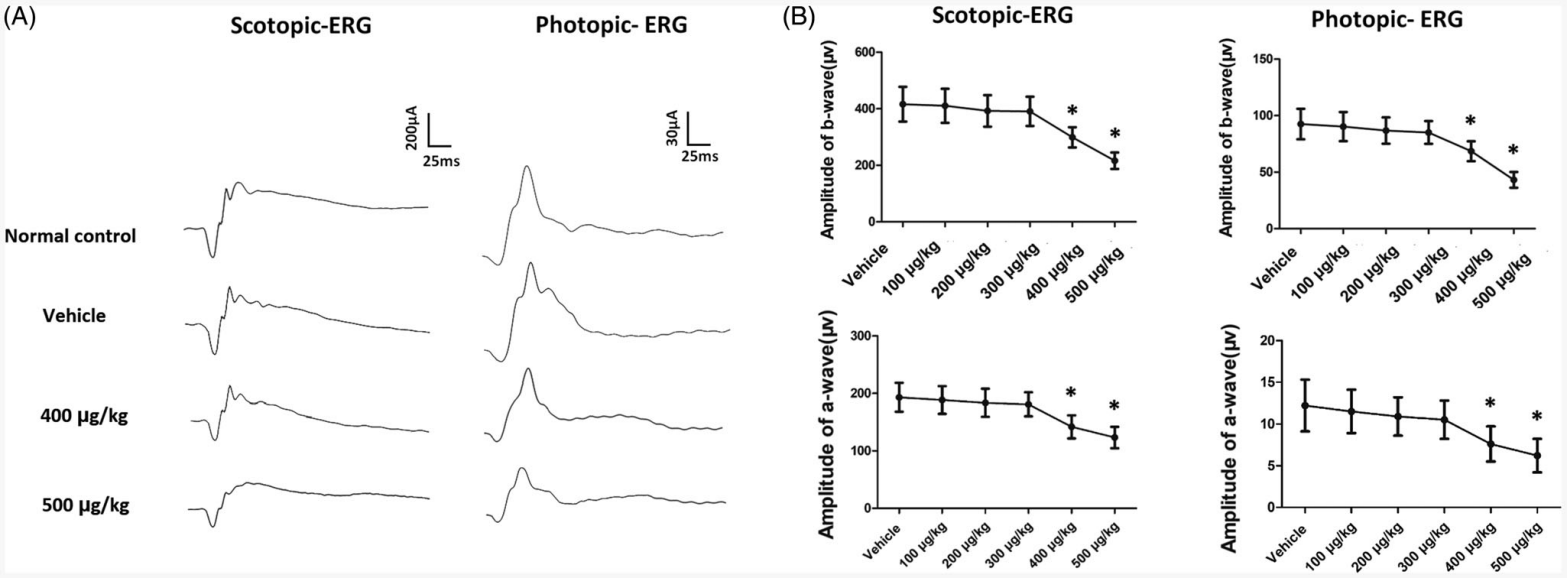
(A) ERG examinations showed that intravitreal melatonin at high dosage would impaire the visual function of mice. (B) The amplitudes of ERG a- and b-wave 400 and 500 µg/kg groups declined significantly compared with normal controls (ANOVA analysis followed by Bonferroni’s post hoc analysis was performed, *p< .05, for differences between groups).

Optokinetic behavioral tests were performed to evaluate the status of visual function. The visual acuity and contrast sensitivity were not significantly different between the vehicle, 100, 200, and 300 µg/kg groups (*p*  > .05; *n* = 10; Figure 2(C)). The visual acuity and contrast sensitivity of the 400 and 500 µg/kg groups declined significantly compared with normal controls (*p* < .01; *n* = 10; Figure 2(D)). In greater detail, these optokinetic parameters were smaller in the 500 µg/kg than that in the 400 µg/kg group (*p* < .01; *n* = 10). These findings suggested that intravitreal melatonin at high dosage yielded deleterious effects on the optokinetic response. The amplitudes of ERG a- and b-wave in the vehicle, 100, 200, and 300 µg/kg groups were not significantly different from those of normal controls (*p* > .05; *n* = 10; Figure 3(A)). The mice in the 400 µg/kg group showed 28.2% reduction in the amplitude of scotopic b-wave, and 25.9% decrease in the amplitude of photopic b-wave, respectively (Figure 3(B)). The impairments were more severe in the 500 µg/kg group: the scotopic and photopic b-wave amplitudes in the 500 µg/kg decreased by ∼51.9% and ∼52.5% compared to normal control group. The scotopic and photopic a-wave amplitudes in the 400 µg/kg decreased by ∼26.7% and ∼37.8% compared to normal control group. The scotopic and photopic a-wave amplitudes in the 500 µg/kg decreased by ∼36.2% and ∼49.2% compared to normal control group. These data agreed well with the histological results. Different components of ERGs waveforms can reflect the status of visual pathways, with a-wave represents the response of photoreceptors and b-wave represents the electrophysiological activities of bipolar cells (Robson & Frishman, 2014). These findings suggest that intravitreal melatonin at high dosage would affect the visual function of mice.

### Intravitreal delivery of melatonin impaired the cone photoreceptors

**Figure 4.**
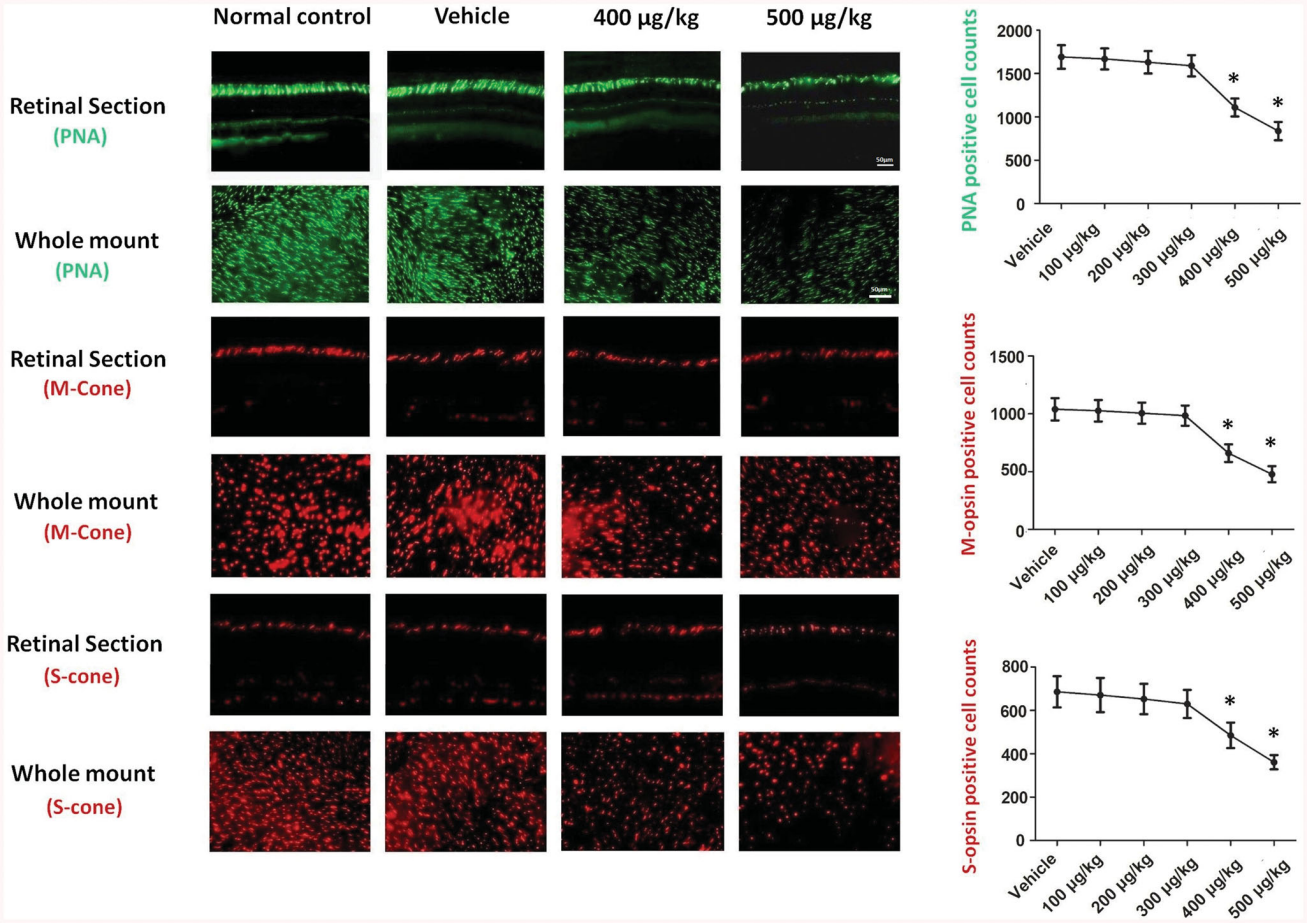
Immunostaining was performed to evaluate the survival rate of cone photoreceptors. The PNA staining in the 400 and 500 µg/kg groups decayed after melatonin delivery. The M- and S-cone photoreceptor counts in the 400 and 500 µg/kg groups decreased significantly compared with normal controls, suggesting that the cone photoreceptors would be impaired by the high dosage of intravitreal melatonin (ANOVA analysis followed by Bonferroni’s post hoc analysis was performed, *p< .05, for differences between groups).

Immunostaining was performed to evaluate the survival rate of cone photoreceptors after melatonin delivery. Extensive punctate PNA staining was detected in the retinal specimens of vehicle, 100, 200, and 300 µg/kg groups. As shown in these immunostaining works, the PNA staining in the 400 and 500 µg/kg groups decayed after melatonin delivery (Figure 4). The PNA-positive cells in retina whole-mounts were quantified. The number of PNA-positive cells in the 400 and 500 µg/kg groups declined significantly (*p* < .01; *n* = 10), while those in the 100, 200, and 300 µg/kg groups did not differ significantly from normal controls (*p* > .05; *n* = 10). The PNA-positive cell counts in the 400 and 500 µg/kg groups decreased by ∼34.5% and ∼50.6% compared to normal controls. Furthermore, the viability of cone photoreceptors was assessed using opsin antibodies. The M-cone photoreceptor counts in the 400 and 500 µg/kg groups decreased by ∼35.8% and ∼52.2% compared with normal controls. The S-cone photoreceptors counts in the 400 and 500 µg/kg groups decreased by ∼29.5% and ∼47.0% compared with normal controls. Conversely, the S- and M-cone photoreceptors counts were not significantly different between the 100, 200, 300 µg/kg groups and normal control group (*p* > .05; *n* = 10), suggesting that the cone photoreceptors would be impaired by the high dosage of intravitreal melatonin. Since there is a gradient in the distribution of S *vs.* M cones in the rodent retina (Haverkamp et al., 2005), the number of cones in each retinal quadrant surrounding the ONH was compared among the animal groups (Table 1).

**Table 1.** Cell counts in different quadrants of retina whole mounts.

Location	Vehicle	100 μg/kg	200 μg/kg	300 μg/kg	400 μg/kg	500μg/kg
PNA-positive cell counts						
S T	1733 ± 122	1701 ± 123	1693 ± 129	1631 ± 125	1026 ± 115*	728 ± 99*
IT	1711 ± 129	1680 ± 122	1661 ± 116	1639 ± 113	1238 ± 106*	962 ± 101*
C	1682 ± 125	1662 ± 109	1641 ± 128	1619 ± 108	1093 ± 102*	806 ± 95*
IN	1662 ± 116	1632 ± 126	1617 ± 113	1582 ± 121	1201 ± 95*	902 ± 103*
SN	1689 ± 118	1638 ± 118	1626 ± 126	1558 ± 113	1002 ± 98*	758 ± 91*
Average	1692 ± 123	1669 ± 121	1630 ± 129	1590 ± 122	1109 ± 103*	837 ± 106*
S-opsin positive cell counts						
S T	326 ± 41	318 ± 39	305 ± 36	301 ± 38	214 ± 29*	169 ± 23*
IT	539 ± 45	531 ± 50	525 ± 48	513 ± 41	388 ± 39*	306 ± 31*
C	838 ± 56	826 ± 66	813 ± 61	809 ± 55	696 ± 51*	582 ± 50*
IN	889 ± 61	867 ± 72	861 ± 60	857 ± 53	730 ± 52*	659 ± 53*
SN	823 ± 68	815 ± 61	798 ± 53	789 ± 51	508 ± 45*	445 ± 45*
Average	686 ± 72	671 ± 70	653 ± 70	630 ± 65	485 ± 59*	361 ± 38*
M-opsin positive cell counts						
S T	1189 ± 83	1172 ± 86	1136 ± 81	1123 ± 89	801 ± 78*	619 ± 54*
IT	1155 ± 81	1125 ± 87	1108 ± 79	1082 ± 79	621 ± 72*	409 ± 51*
C	1140 ± 94	1126 ± 94	1096 ± 90	1076 ± 90	645 ± 65*	563 ± 56*
IN	1132 ± 76	1133 ± 90	1121 ± 84	1089 ± 84	583 ± 56*	419 ± 50*
SN	1165 ± 82	1163 ± 82	1145 ± 88	1091 ± 88	782 ± 71*	536 ± 52*
Average	1163 ± 96	1142 ± 93	1126 ± 91	1095 ± 88	683 ± 76*	509 ± 69*

*p< .05 for difference compared with control group. All values are presented as mean ± SD; *n* = 10 per group.

### Regional response after intravitreal delivery of melatonin

**Figure 5.**
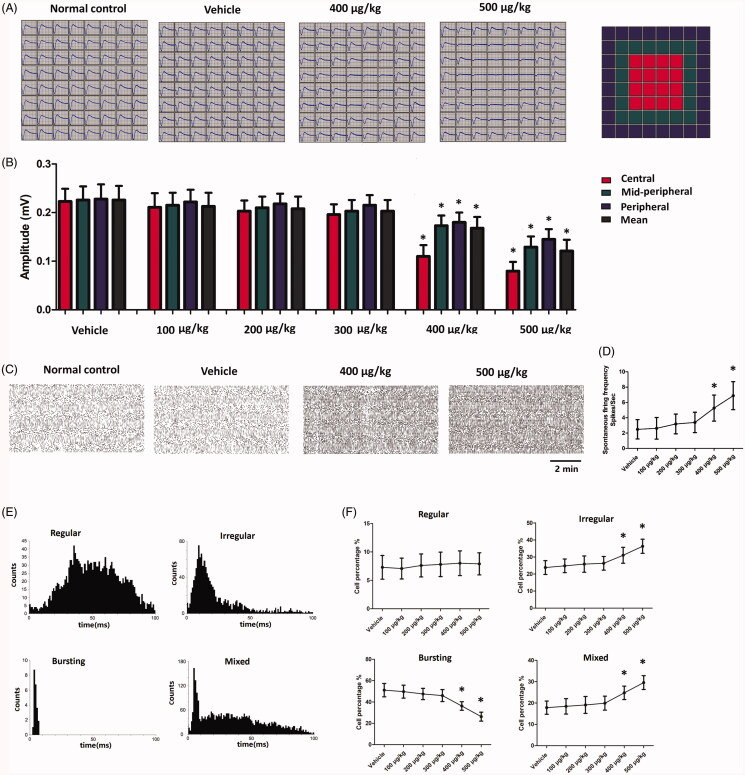
(A) The MEA was divided into three regions according to their distances to ONH. (B) The mean LFPs amplitudes of 400 and 500 µg/kg group declined significantly after melatonin delivery. In particular, the LFPs were more severely impaired in the central region than those in the peripheral regions. (C) The spiking analysis showed that spontaneous firing frequency increased markedly in the 400 and 500 µg/kg groups. (D) The spontaneous firing frequency in the 400 and 500 µg/kg groups increased significantly. (E) The RGCs could be classified into the bursting cells, regularly firing cells, irregularly firing cells, and mixed type cells. (F). The percentages of bursting and mixed-type firing RGCs were significantly higher in the 400 and 500 µg/kg groups compared with normal controls. The percentage of irregularly firing RGCs was significantly lower in the 400 and 500 µg/kg group compared with normal controls (ANOVA analysis followed by Bonferroni’s post hoc analysis was performed, *p< .05, for differences between groups).

**Figure 6.**
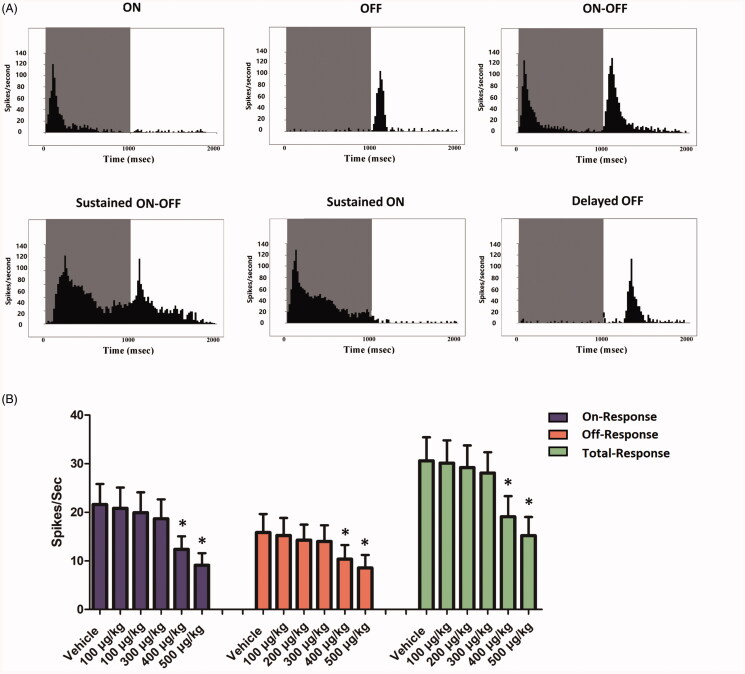
(A) The post-stimulus time histogram (PSTH) and the raster plots of individual units were used to categorize the light-induced responses of RGCs. (B) Both the ON- and OFF-response were affected by high dose intravitreal melatonin. In particular, the ON-response was more severely affected by high dose intravitreal melatonin (ANOVA analysis followed by Bonferroni’s *post hoc* analysis was performed, *p< .05, for differences between groups).

MEA recording was carried out to assess the electrical activity of regional retina. The electrical array was divided into three regions according to their distances to ONH: the central, the mid-peripheral, and the peripheral regions (Figure 5(A)). The mean LFPs amplitudes of vehicle, 100, 200, and 300 µg/kg groups were not significantly different from that of normal controls (*p* > .05; *n* = 10). Compared with normal controls, the mean LFPs amplitudes of 400 and 500 µg/kg group declined significantly after melatonin delivery (*p* < .01; *n* = 10; Figure 5(B)). The mean LFPs amplitudes of 400 and 500 µg/kg group were, respectively, ∼25.7% and ∼46.5% of normal controls. Topographic analysis showed that the LFPs amplitudes of central, the mid-peripheral, and peripheral region decreased by ∼32.3%,∼23.5%, and ∼19.3% in the 400 µg/kg group, and ∼54.0%,∼43.5%, and ∼36.5% in the 500 µg/kg group. These findings suggested that photoreceptors activity was more severely impaired in the central region than that in the peripheral regions. Furthermore, the spiking analysis was performed to study the firing activities of RGCs (Figure 5(C)). The spontaneous firing frequency in the vehicle, 100, 200, and 300 µg/kg groups were not significantly different from that of normal control group (*p* > .05; *n* = 10). The spontaneous firing frequency in the 400 and 500 µg/kg groups increased significantly (*p* < .01; *n* = 10; Figure 5(D)). The spontaneous firing frequency increased by ∼111.2% and ∼176.3.0% in the 400 and 500 µg/kg groups, respectively. The majority (92.8%) of the detected RGCs could be classified into the bursting cells, regularly firing cells, irregularly firing cells, and mixed type cells (Figure 5(E)). The percentages of bursting and mixed-type firing RGCs were significantly higher in the 400 and 500 µg/kg groups compared with normal controls (*p* < .01; *n* = 10; Figure 5(F)). On the other hand, the percentage of irregularly firing RGCs was significantly lower in the 400 and 500 µg/kg group compared with normal controls (*p* < .01; *n* = 10). The percentage of regularly firing RGCs in the 400 and 500 µg/kg group was not significantly different from normal controls (*p* > .05; *n* = 10). The PSTHs and the raster plots of individual units were used to categorize the light-induced responses of RGCs (Figure 6(A)). We found that the ON response was more severely affected by high dose intravitreal melatonin (Figure 6(B)). These findings suggest that intravitreal delivery of high dosage melatonin would affect the electrical activity of RGCs.

## Discussion

Melatonin is as a ubiquitously distributed molecule with robust therapeutic potentials. The synthesis pattern of melatonin and its contributing role in biological clock processes have been well characterized (Aranda et al., 2017). An emerging feature that characterizes melatonin is the capacity to modulate the physiology and biology of retinal neurons. Melatonin shows robust potency to detoxify the free radicals and oxygen derivatives, thereby improving the homeostasis of retinas (Blasiak et al., 2016). In ophthalmological experiments, melatonin can be delivered through the intravitreal route to exert its therapeutic actions (García-Caballero et al., 2018; Li et al., 2019). However, the exact pharmacokinetic and toxic aspects for this delivery approach remain to be clarified. In this study, we find that intravitreal delivery of high dosage melatonin (400–500 µg/kg) would destroy the retinal morphology and impair the visual function of mice. Conversely, the melatonin administration at lower dose (100–300 µg/kg) does not have any significant effects on the retinas. In these mice exposed to higher dose melatonin, the retinal architecture is destroyed and the retinal neurons are lost in some regions. These findings highlight that intravitreal melatonin is not always beneficial, especially when it is administered at high dosage. The mechanism underlying these toxic effects should be ascribed to the activation of phagocyte system (Weiss, 1989; Laurent et al., 2017). As a lipophilic molecule, melatonin can cross instantly the blood–retina barrier and enter the subcellular compartments. The ocular melatonin level would increase instantly after intravitreal delivery (Shida et al., 1994). It is shown that high dose melatonin can disturb the retina homeostasis and act as chemoattractant for the activated phagocytes (e.g. neutrophils and macrophages). These activated phagocytes release cytokines and inflammation mediators into the cytoplasm which would subsequently destroy the retinal tissue (Ashander et al., 2019). Moreover, the membranes of retinal photoreceptors contain abundant polyunsaturated fatty-acids vulnerable lipid peroxidation (Prokopiou et al., 2018). Phagocytes can produce diverse superoxide radicals and oxidant species when they are exposed to exogenous stimulus. In particular, the severity of oxidative damage is closely correlated to the degree of inflammation (Sarangarajan & Apte, 2005; Rendra et al., 2019). It is well known that the visual pigment (e.g. rhodopsin) in the outer segment of photoreceptors can sensitize the formation of singlet oxygen (van Norren & Vos, 2016). Notably, melatonin inhibits the release of dopamine and increases the light sensitivity of photoreceptors (Dubocovich, 1983). Excessive exposure of photoreceptors to light stimulus would cause deleterious lipid peroxidation and irreversible visual impairments. Previous studies have demonstrated that inappropriate exposure of retina to melatonin would be detrimental to photoreceptor survival, as supported by the fact that melatonin increases the susceptibility of photoreceptors to light-induced damages (Wiechmann et al., 2008). In particular, there is a risk of melatonin accumulation as the result of multiple treatments. On the other hand, intravitreal injection of luzindole, a melatonin receptor competitive antagonist, can effectively protect photoreceptors from phototoxic insults (Sugawara et al., 1998). This may explain the fact that retinal damages and visual impairments occurred during the high dose melatonin administration. Müller cells constitute the main glial cells of the retina (Eastlake et al., 2020). They are spatially distributed across retinal laminations, facilitating their close membrane interactions with different types of retinal neurons (Di Pierdomenico et al., 2020). Müller cells are characterized by their active metabolic functions, and can become reactive under pathological conditions, leading to their production of inflammatory and neurotoxic factors (Pfeiffer et al., 2016). Moreover, Müller cells are sensitive to metabolic alterations under retinal stress, and respond to photoreceptor death by undergoing reactive gliosis (Hamon et al., 2017). Thus Müller cells might be involved in the retinal degeneration after the intravitreal delivery of high dose melatonin. Further studies are needed to verify the exact role of Müller cells during the retinal degeneration.

Due to the complex anatomy and biological barriers of the eye, drug delivery to the retina constitutes an enormous challenge for pharmaceutical trials (Varela-Fernández et al., 2020). Intravitreal injection is a minimal invasive approach to deliver therapeutic agents to the posterior eyeball. This delivery mode has been widely applied to treat ocular diseases including endoophthalmitis, IOP elevation, angiogenesis, and neurodegeneration (Melo et al., 2020). Direct intravitreal delivery has the prominent advantage of achieving immediate high drug concentrations in the vitreous humor, avoiding these systemic side effects on unwanted organs (De Stefano et al., 2011). However, not all the side effects are completely eliminated and the retinal toxicity is recognized as a critical complication for intravitreal delivery. In this study, intravitreal melatonin causes toxic effects on retinal neurons, implicating that this delivery route might be inappropriate for high dose melatonin. On the other hand, intravitreal injection of low dose melatonin would not destroy the retina architecture, or disturb the visual function of mice. Accordingly, it is desirable to develop novel controlled release carriers which can stabilize the melatonin concentration in the vitreous, thereby minimizing the toxic effects on retinas.

Abundant research evidences suggest that exogenous melatonin exerts beneficial effects on the RGCs (Juybari et al., 2019). However, a previous study also shows that intravitreal injection of high dose melatonin results in RGCs loss in the guinea retina (Yilmaz et al., 2004). Our results confirm and extend the previous study that high dose melatonin would impair the RGCs survival and affect their electrophysiology activities. RGCs constitute the innermost layer of the retina. They integrate visual information from photoreceptors and project these signals (firing spikes) to the brain *via* the optic nerve (Wienbar & Schwartz, 2018). RGCs project their 1.2 million axons, composing the optic nerve, to the brain, transmitting the visual signals gathered by the retina, ultimately leading to formed vision in the visual cortex (Maresca et al., 2013; Carelli et al., 2017). Loss of RGCs is a common feature of optic neuropathies (Adornetto et al., 2020). It has been reported that the combined use of melatonin and Zoloft can result in the melatonin/dopamine imbalance in retina, manifesting as a toxic optic neuropathy (Lehman & Johnson, 1999). Since the signals from the inner retina will affect the circadian activities of retinal photoreceptors, we use the MEA to assess the visual signal of inner retina after melatonin delivery. MEA is an excellent platform for testing therapeutics or toxic effects of pharmacological agents. It is also applied to study the spontaneous activity of neuronal and cardiac cell populations (Andrew et al., 2010). This system can simultaneously record the extracellular signals from multiple sites of circuits in real time, increasing spatial detection and thereby providing a robust evaluation of network function (Bruno et al., 2020). The key advantage of this technique is the potency to detect and excite large populations of neurons, without causing adverse effects to the tissue. MEA is sensitive to detect the delicate changes of retinal network, and evaluate accurately the statement of visual function (Neumann et al., 2008). MEA detects a pronounced increase of the spontaneous firing frequency in these mice received high dose melatonin. Accordingly, excessive intravitreal melatonin would impair the RGCs’ capacity to encode visual signals. Overexposure of melatonin may be hazardous for the visual signal transmission of mice retinas. Moreover, MEA recording provides some topographic information about the vitality of photoreceptors. The photoreceptors in the central retina are more severely affected by intravitreal melatonin. Generally, photoreceptors in the central retina are more vulnerable to toxic or pathogenetic factors than those in the peripheral region (Stone et al., 1999; Homma et al., 2009). The retina is a highly organized neural tissue consisting of three neural layers and two synaptic layers. Vascular system that nourish the mouse retina mirror this organization consisting of three plexus layers, or plexuses, that run parallel within the retina, connected by interplexus vessels to create a closed vascular network (Simmons & Fuerst, 2018). The dependence of the retina on its blood supply makes it highly vulnerable to any pathological factors and indeed ocular diseases (Ramos et al., 2013). In the mouse retinal, the blood supply is much more abundant in the peripheral region than that in the central region (areas closest to the ONH) (Robert & Zhang, 2017; Ye & Smith, 2018; Reiner et al., 2018). Therefore, excessive melatonin in the peripheral retina can be eliminated rapidly by potent blood circulation.

Loss of cone photoreceptors is a pivotal event in the development of severe vision impairment. Melatonin is correlated intimately with the cone photoreceptor viability during aging (Wiechmann & Sherry, 2013). Normally, the amount of melatonin synthesized by the photoreceptor is much smaller than that by the pineal gland, and the retinal melatonin level is maintained at extremely low in the presence of light (Felder-Schmittbuhl et al., 2018). Such strict control of retinal melatonin level implies that excessive melatonin supplements may be deleterious for the cone photoreceptors. In the rodent retinas, cones represent a minor percentage (approximately 3%) of the total number of photoreceptors with the M-cones being the more prevalent type and with an extensive distribution, while the S-cones are low in number and with an uneven distribution gradient (Gianesini et al., 2016). In this study, PNA marker and opsin antibodies are used to determine the cone vitality. The M- and S-cone types were impaired by the high dose melatonin to a similar extent. These results provide valuable baseline data for future researches studying the intravitreal delivery of melatonin. The toxic effects on cone vitality would be a highly relevant factor when considering both the timing and dosage of melatonin.

In summary, intravitreal delivery may expose the retina to excessive melatonin, resulting in detrimental effects on the retinal morphology and visual function. In light of the evidence presented in this study, we suggest that it will be prudent to avoid the intravitreal-administration of high dose melatonin. The toxic effects induced by the intravitreal delivery of melatonin raises new demands regarding the controlled release of exogenous melatonin in the eye.
